# Supplementary material for: Prevalence of neutralising antibodies against SARS-CoV-2 in acute infection and convalescence: A systematic review and meta-analysis
Source: PLoS Negl Trop Dis. 2021 Jul 8;15(7):e0009551. doi: 10.1371/journal.pntd.0009551 (PMC8291969; doi:10.1371/journal.pntd.0009551)
Supplement: S1 Table — (DOCX) [file pntd.0009551.s001.docx]

**S1 Table. Full Search Strategy.**

| **Medline and Cochrane Searches** | |
| --- | --- |
| **Keyword (linked using OR)** | **MeSH (linked using OR)** |
| “severe acute respiratory coronavirus 2”  “SARS-CoV-2”  sars AND virus  sars AND cov  “COVID-19” “COVID 2019”  “novel coronavirus”  “new coronavirus”  “Wuhan coronavirus”  “Coronavirus disease 19”  “2019-nCoV” | Betacoronavirus  Coronavirus Infections  SARS Virus  Severe Acute Respiratory Syndrome  Middle East Respiratory Syndrome Coronavirus |
| **Keyword (linked using OR)** | **MeSH (linked using OR)** |
| “neutralising antibod*” “neutralizing antibod*” neutralising AND antibod* neutralizing AND antibod* | Broadly Neutralizing Antibodies  Antibodies, Neutralizing |
| **Web of Science search** | |
| ALL = (“severe acute respiratory coronavirus 2” OR “SARS-CoV-2” OR (sars AND virus) OR (sars AND cov) OR “COVID-19” OR “COVID 2019” OR “novel coronavirus” OR “new coronavirus” OR “Wuhan coronavirus” OR “Coronavirus disease 19” OR “2019-nCoV”) | |
| ALL = (“neutralising antibod*” OR “neutralizing antibod*” OR (neutralising AND antibod*) OR (neutralizing AND antibod*) ) | |
| Combine above with AND | |
| **Medrxiv and Biorxiv searches** | |
| **Keyword (linked using OR):**  “neutralising antibod*”  “neutralizing antibod*”  neutralising AND antibod*  neutralizing AND antibod* | |
| Papers then selected for relation to SARS-CoV-2 | |
| **Collabovid.org search (**<https://www.collabovid.org/>) | |
| **Keyword (linked using OR):**  “neutralising antibod*”  “neutralizing antibod*”  neutralising AND antibod*  neutralizing AND antibod* | |
