# Supplementary material for: Prevalence of neutralising antibodies against SARS-CoV-2 in acute infection and convalescence: A systematic review and meta-analysis
Source: PLoS Negl Trop Dis. 2021 Jul 8;15(7):e0009551. doi: 10.1371/journal.pntd.0009551 (PMC8291969; doi:10.1371/journal.pntd.0009551)
Supplement: S1 Text — (DOCX) [file pntd.0009551.s003.docx]

**S1 Text**

**STATISTICAL ANALYSIS**

1. **Combining prevalences across studies**

Let Yi denote the number of individuals who tested positive for neutralising antibodies (nAB) out of ni tested patients from the i-th study.

Conditionally on random effects Zi, we assume that Yi is a Binomial distribution, with probability of a positive test pi which model as logit-linear regression which takes the form

                              logpi1-pi=+Zi                                       (1)

where is an intercept parameter. We assume that Ziare a set of independent and identically distributed Gaussian variables with mean 0 and variance 2.

To fit the model in (1), we use the maximum likelihood method. We approximate the likelihood function using the Quasi Monte Carlo approximation based on 1000 points generated from the Halton sequence.

To combine estimates across studies, we first generate 10,000 samples from the distribution of pi conditionally on the data Yi=yi, using the inverse transformation method. For each of the 10,000 simulations, we average the pi across studies by conditioning on the different variables reported in the row of Table 2 of the main manuscript. The resulting 10,000 samples of averaged prevalences, are summarized through their mean and the quintiles 0.025 and 0.975 as reported in Table 2.

1. **Bivariate modelling of IgG and nAB prevalence across studies**

Using a similar notation to that of the previous section, let Yij denote the number of individuals who tested positive for antibody j={nAB, IgG}out of nij tested patients from the i-th study.

Conditionally on random effects Zij, we assume that Yij is a Binomial distribution, with probability of a positive test pij which model as logit-linear regression which, for j=IgG, takes the form

logpi,IgG1-pi,IgG=di'IgG+Zi,IgG

where di is a vector of covariates corresponding to titre cut off and sampling setting.

For j=nAB, the linear predictor for the log-odds takes the form

logpi,nAB1-pi,nAB=di'nAB+ei'+Zi,nAB

where ei is a vector of dummy variables indicating the methods used for the detection of the neutralizing antibody.

Finally, we assume that  Zi,IgG and Zi,nAB are random Gaussian variables, each with variance 2 and correlation, which corresponds to the correlation between antibodies after adjusting for sampling setting, titre cut off and methods of detection of the neutralizing antibody.

We fit the model using a Quasi-Monte Carlo method for maximum likelihood estimation. The 95% confidence intervals of the model parameters are obtained via-parametric bootstrap. All the code developed in R software environment is made available in the supplementary material.

1. **Bivariate modelling of individual IgG and nAB concentrations**

We carry the analysis of the individual-level data for each study separately.

Let Yij* denote the concentration of the antibody j={nAB, IgG} for the i-th patient.

We then assume that the log{Yi,nAB*} and log{Yi,IgG*} are correlated Gaussian distributions with means

                              logij=dijTj+Uijj, for j={nAB, IgG}                  (1)

where  Ui,IgG and Ui,nAB are a pair of Gaussian distributions with mean 0 and variance IgG2 and nAB2, respectively, and correlation. We fit the model using the maximum likelihood method.

Due to the heterogeneity in information reported from the different studies, the covariates dij differ for the different analysis. We report these covariates, including their estimates and confidence intervals in Table 2A.
