# Supplementary material for: Prevalence of neutralising antibodies against SARS-CoV-2 in acute infection and convalescence: A systematic review and meta-analysis
Source: PLoS Negl Trop Dis. 2021 Jul 8;15(7):e0009551. doi: 10.1371/journal.pntd.0009551 (PMC8291969; doi:10.1371/journal.pntd.0009551)
Supplement: Attachment — Submitted filename: PNTD-D-21-00266 review.docx [file pntd.0009551.s004.docx]

Manuscript title: Prevalence of neutralizing antibodies against SARS-CoV-2 in acute infection and convalescence: a systematic review and meta-analysis

Manuscript ID: PNTD-D-21-00266

Summary: Savage et al., has conducted a systematic review and meta-analysis to identify the proportion of individuals possess neutralizing titers against SARS-CoV-2. Authors have used three models using the maximum likelihood method and extracted 27 published studies to do meta-analysis. Overall, Savage et al., identified that 85% of individuals had detectable neutralizing antibodies against SARS-CoV-2 infection and this varies based on severity of infection.

Comments to authors:

Manuscript was well written, with a goal to search for proportion of individuals with SARS-CoV-2 neutralizing antibodies. Authors have done extensive literature search, extracted, summarized each study well and analyzed data from the studies that assessed the neutralizing antibody titers against SARS-CoV-2 virus. Conclusions drawn from the analysis were clear. But I, have few concerns outlined below to be addressed by the authors:

Major comments:

It will enhance the manuscript if authors could state what additional knowledge has been gained by doing meta-analysis compared to all those individual studies included in this study.

It is expected to have some degree of error in each study, as authors stated every study used different method or assay to estimate neutralizing antibodies then why do meta-analysis

Are authors trying to test their statistical models? If so, what is the rationale behind using only this model?

Minor comments

Results:

1. I recommend authors to clarify to readers in section “Correlation of neutralizing and IgG antibodies” whether IgG antibodies are total serum IgG’s or IgG’s specific against SARS-CoV-2 spike protein.
2. quasi-Monte Carlo is a derandomized algorithm will the results differ using randomized quasi-Monte Carlo algorithm
