# Supplementary material for: Prevalence of neutralising antibodies against SARS-CoV-2 in acute infection and convalescence: A systematic review and meta-analysis
Source: PLoS Negl Trop Dis. 2021 Jul 8;15(7):e0009551. doi: 10.1371/journal.pntd.0009551 (PMC8291969; doi:10.1371/journal.pntd.0009551)
Supplement: Attachment — Submitted filename: Reply_PNTD_R1.docx [file pntd.0009551.s005.docx]

**To:**

**Tao Lin, DVM, MSc**

**Associate Editor**

**PLOS Neglected Tropical Diseases**

**Liesl Zuhlke**

**Deputy Editor**

**PLOS Neglected Tropical Diseases**

**Revised Manuscript #PNTD-D-21-00266**

Dear Editors,

We would like to thank the referee for his/her constructive comments and his/her recommendation of acceptance of the manuscript after changes.

We have made the revisions suggested and have prepared an itemized reply to all issues raised below, as requested.

We hope these replies are satisfactory to the reviewers and your office. However, please contact us again if you feel there are issues that were not addressed appropriately.

We look forward to your decision.

Best regards,

Luis E. Cuevas

On behalf of all authors.

**Reviewer's Responses to Questions**

**Methods**

Reviewer #1: The study is clearly articulated, and the objectives well described. The design is appropriately set for a study that is needed in the field. The studies selected are often small and a larger size would benefit however the analysis provides clear conclusions using well established methodology.

Re: Thank you for your comment. No action is required.

**Results**

Reviewer #1: The analysis presented match the analysis plan. The neutralizing antibody results are clearly and well presented. The IgG analysis comes with a lot of complexity is lacking granularity.

Re: No specific action is requested.

**Conclusions**

Reviewer #1: The conclusions are supported great deal by the data presented and the limitations of analysis are described. The authors discuss how these data are helpful to advance our understanding of the neutralizing antibody immunity against SARS-CoV2. However, larger studies are needed to be able to address the relevance for public health recommendations.

Re: No action is required.

**Editorial and Data Presentation Modifications**

**Reviewer #1:**

**Results**

The IgG/nAntibodie association analysis needs differentiation between different viral antigen or limit it to the surface antigen against which the neutralizing antibodies are directed against.

Re: We have clarified this aspect. Please, see:

“The modelled correlation between the aggregate proportion of patients with measurable IgG against SARS-CoV-2 and neutralising antibodies was very low (0.055 or poor correlation)”.

**Discussion**

“The lack of neutralising antibodies in a small but significant group of patients might be explained by some patients having responses confined to other SARS-CoV-2 antigens or mediated through T cells, which are not detected by the neutralisation assays in this meta-analysis.” It is not clear who those groups of patients are.

Re: We agree with the referee’s statement. We have reworded as:

“The lack of neutralising antibodies in a small but significant group of patients seen in this review might be explained by some patients having responses confined to SARS-CoV-2 antigens not identified by the assays used, or mediated through T cells, which are not detected by the neutralisation assays in this meta-analysis.”

“Mild infections may also elicit responses that are restricted to the mucosal cells, where defence responses are dominated by the secretory immune system.” Interesting statement and good hypothesis that is not evidenced.

Re: We have added a reference to this statement.

“The proportion of participants with neutralising antibodies in the second month was slightly higher among plasma donors and slightly lower among patients recruited from hospital. However, the differences were small and confidence intervals overlapped. Moreover, neutralising antibodies peak within 21 days after symptoms onset and the one month is not ideal to demonstrate time differences” Do you mean that antibodies in hospitalized individuals might decline faster? Do the responses peak within 21 days for both groups?

Re: What we mean is that the cut off of 21 days is really too close to the end of the first month. As the start of the disease is often poorly defined, this cut off may misclassify patients. Reworded as:

“The proportion of participants with neutralising antibodies in the second month was slightly higher among plasma donors and slightly lower among patients recruited from hospital. However, the differences were small and confidence intervals overlapped. As neutralising antibodies peak within 21 days after symptoms onset, and symptoms onset if often poorly documented, it may be that the one-month timepoint is not ideal to demonstrate differences over time.”

**Summary and General Comments**

Reviewer #1: The authors describe a meta-analysis of published studies describing the neutralising antibody titres induced by SARS-CoV 2 acute infection. This is an important study considering the need to understand corelates of immunity by both infection and vaccination. Furthermore, increasing evidence is arguing for the role of neutralizing antibodies. One element that is clearly understood throughout the study is the lack of large cohort comprehensive studies to establish this fact. Only very few studies could be retained for the analysis amongst large number of publications initially selected. None the less the analysis is well conducted, and the authors conclude that the disease severity is largely correlating with the levels of neutralizing antibodies induced.

There are differences between neutralization assay sensitivity that also show throughout the study and it is unclear to what degree it will affect the analysis.

The authors have investigated the associations between neutralizing antibody titres and the Elisa IgG titres. This part of the study has some weakens as besides the neutralising antibody assay variation the binding antibody assays can also be very variable. The type of assay used and most of all the origin of recombinant antigen utilised will influence the assay a great deal. In addition, table 1 indicates a variety of viral antigens utilised to measure IgG and the study lacks granularity.

Re: We accounted the variation in methodology between studies by modelling individual level data for each study (not across studies). However, we still observed a large variation in the correlations obtained for each study. This reinforces that those with previous infection may not show immunity on current testing methodologies and how this correlates with protection against reinfection is unknown.

**Comments to authors:**

Manuscript was well written, with a goal to search for proportion of individuals with SARS-CoV-2 neutralizing antibodies. Authors have done extensive literature search, extracted, summarized each study well and analyzed data from the studies that assessed the neutralizing antibody titers against SARS-CoV-2 virus. Conclusions drawn from the analysis were clear. But I, have few concerns outlined below to be addressed by the authors:

Major comments:

It will enhance the manuscript if authors could state what additional knowledge has been gained by doing meta-analysis compared to all those individual studies included in this study.

It is expected to have some degree of error in each study, as authors stated every study used different method or assay to estimate neutralizing antibodies then why do meta-analysis.

Re:

“In the event of a new pandemic virus there is a limited window where the natural epidemiology of the virus can be observed prior to treatment or vaccination. Without large cohort studies only, a fragmented picture was available, and ongoing cohorts will need to account for virus variants, treatment and vaccination. Combining studies and performing a meta-analysis allowed an analysis of data from early stages and gives a more complete picture of the natural immunity at the beginning of the pandemic”.

Are authors trying to test their statistical models? If so, what is the rationale behind using only this model?

Re: Our statistician indicates the proposed model provides the most suitable approach to analyse the data. This is due to the model 1) respecting the format of the count data using a Binomial likelihood and 2) accounting for the heterogeneities across studies using suitable covariates and random effects.

**Minor comments**

**Results:**

I recommend authors to clarify to readers in section “Correlation of neutralizing and IgG antibodies” whether IgG antibodies are total serum IgG’s or IgG’s specific against SARS-CoV-2 spike protein.

Re: We have clarified in the text that it was IgG against SARS-CoV-2. However, studies included a variety of proteins, and these were listed in the table.

Quasi-Monte Carlo is a derandomized algorithm will the results differ using randomized quasi-Monte Carlo algorithm

Re: The integrals that we need to approximate were all in 1 dimension. This scenario can be easily dealt using a simple Quasi-Monte Carlo. We acknowledge that more sophisticated approximations could be used, but the differences between these approximations and the standard Quasi Monte Carlo would be negligible.
